# Effects of *Lactobacillus plantarum* ZG-7 on the intestinal barrier and intestinal flora of Muscovy ducks infected with avian pathogenic *Escherichia coli*

**DOI:** 10.3389/fimmu.2025.1701722

**Published:** 2026-01-16

**Authors:** Song Peng, Bilin Xie, Guiheng Mei, Yaxiong Ma, Xin Lin, Mengshi Zhao, Fengqiang Lin, Zhaolong Li

**Affiliations:** 1 Institute of Animal Husbandry and Veterinary Medicine of Fujian Academy of Agricultural Sciences, Fuzhou, China; 2 Putian Institute of Agricultural Sciences, Putian, China; 3 Key Laboratory of Animal Pathogen Infection and Immunology of Fujian Province, College of Animal Sciences, Fujian Agricultural and Forestry University, Fuzhou, China; 4 Key Laboratory of Traditional Chinese Veterinary Medicine and Animal Health in Fujian Province, College of Animal Sciences, Fujian Agriculture and Forestry University, Fuzhou, China

**Keywords:** *Lactobacillus plantarum* ZG-7, avian pathogenic *Escherichia coli*, intestinal barrier, cecal microbiota, Muscovy duck

## Abstract

**Introduction:**

Avian pathogenic *Escherichia coli* (APEC) poses a serious challenge to global poultry production, where it causes enteritis, septicemia, and high mortality, resulting in substantial economic losses. Although antibiotics have been traditionally used to control APEC, the rise of antimicrobial resistance and concerns over drug residues underscore the need for effective and sustainable alternatives. Probiotics have emerged as promising candidates because of their ability to modulate the intestinal microbiota, strengthen host immunity, and preserve epithelial barrier integrity. In this study, we investigated the protective role of *Lactobacillus plantarum* ZG-7 against APEC infection in Muscovy ducks.

**Methods:**

40 one-day-old ducks (equal numbers of males and females) were randomly allocated to five groups (n = 8 per group): control (CON), probiotic alone (LP), APEC-infected (EC), probiotic-pretreated APEC-infected (LPEC), and colistin sulfate-treated APEC-infected (CSEC), with the latter serving as a positive control and exhibiting expected protective effects. On day 7, ducks in the EC and LPEC groups received two oral doses of pathogenic *E. coli* O78 (3 × 10^9 CFU/mL, 0.2 mL) at an 8-hour interval, while ducks in the CON and LP groups received sterile saline. Serum and intestinal samples were collected on day 15.

**Results:**

APEC infection significantly reduced average daily gain during days 9–15 and across the trial. Histopathological analysis showed epithelial disruption, crypt and gland loss, reduced goblet cells, diminished mucus secretion, and decreased expression of tight junction proteins (ZO-1, MUC2, Occludin). In contrast, *L. plantarum* ZG-7 treatment alleviated intestinal injury and restored growth performance. 16S rRNA sequencing further revealed that APEC challenge increased the abundance of unclassified *Lachnospiraceae, Lachnoclostridium, norank RF39 group*, and *Paludicola* (*P* < 0.05), whereas *L. plantarum* ZG-7 treatment reduced these taxa. Moreover, probiotic supplementation alone significantly enriched *Bacteroides* (*P* < 0.001).

**Discussion:**

Taken together, these results demonstrate that *L. plantarum* ZG-7 helps maintain a beneficial microbial composition, protects epithelial barrier function, and mitigates the adverse effects of APEC infection in Muscovy ducks, highlighting its potential as a natural and sustainable alternative for improving poultry health.

## 1 Introduction

Avian pathogenic *Escherichia coli* (APEC) is widely recognized as the principal etiological agent of colibacillosis in poultry, a condition that seriously compromises flock health and production efficiency worldwide (1). Beyond inducing both systemic and localized infections that impair immune competence, APEC also undermines the intestinal epithelial barrier and disrupts gut microbial ecology. Ducks are particularly vulnerable hosts, often exhibiting progressive weight loss, weakness, and eventually systemic failure followed by death. Studies have shown that APEC attaches to intestinal epithelial cells via fimbrial and non-fimbrial adhesins, facilitating colonization and barrier disruption. This process increases epithelial permeability, enabling bacterial translocation into the bloodstream, where diverse virulence factors provoke widespread inflammatory responses (2). In addition, APEC challenge commonly results in dysbiosis, characterized by the overgrowth of pathogenic taxa and the reduction of beneficial commensals. The conventional strategy for controlling colibacillosis has been antibiotic intervention, typically with agents such as colistin sulfate or enrofloxacin (3). However, the growing prevalence of antimicrobial resistance, along with concerns about drug residues in animal products, has eroded confidence in antibiotic-based approaches and highlighted the need for new, sustainable solutions.

Probiotics are generally defined as live microorganisms that, when administered in adequate amounts, confer health benefits to the host (4). Their biological functions extend beyond simple colonization and include reinforcement of epithelial barrier integrity, competitive exclusion of pathogens, production of antimicrobial metabolites, and modulation of host immune responses. These properties make probiotics attractive candidates for the prevention and management of bacterial infections. Among them, *Lactobacillus plantarum* is a highly versatile lactic acid bacterium that occurs widely in diverse ecological niches, including vegetables, fruits, legumes, dairy and meat products, and beverages, as well as in mucosal environments of humans and animals such as the oral cavity, gastrointestinal tract, and reproductive tract (5). Due to its broad physiological capabilities and well-documented health-promoting properties, *L. plantarum* has been extensively applied in the food industry, clinical practice, and animal production.

Accumulating evidence has demonstrated the beneficial effects of *L. plantarum* on intestinal health. Oral supplementation has been shown to increase the ratio of Firmicutes to Bacteroidetes, enhance microbial diversity, suppress pathogenic bacterial activity, and ameliorate colitis-associated dysbiosis in mice (6). In TNBS-induced colitis, *L. plantarum* LC27 upregulated the expression of tight junction proteins, decreased serum aminotransferase activity, and reduced the relative abundance of *Proteobacteria* and *Bacteroidetes*, thereby restoring intestinal homeostasis (7). In poultry, dietary *L. plantarum* B1 supplementation improved growth performance, reduced cecal *Escherichia coli* counts, increased *Lactobacillus* abundance, and enhanced intestinal mucosal immunity in broilers challenged with *E. coli* K88 (8). Likewise, administration of *L. plantarum* LP-BL0111 markedly decreased diarrhea scores in broilers, modulated gut microbial composition, and strengthened mucosal barrier function, leading to a significant attenuation of *Salmonella*-induced intestinal injury, with improvement rates reported up to 86.45% (9).

Our previous work further demonstrated that the culture supernatant of *L. plantarum* ZG-7 exerted potent inhibitory effects against *Riemerella anatipestifer* by disrupting membrane integrity, thereby suppressing bacterial proliferation (10). These findings, together with extensive evidence from other *L. plantarum* strains, suggest that targeted probiotic supplementation can help establish beneficial microbial communities, preserve intestinal homeostasis, enhance epithelial barrier integrity, and support host growth and immune function. Consequently, *L. plantarum* has attracted considerable attention as a natural alternative for improving poultry health and mitigating bacterial infections.

Despite these advances, the strain-specific probiotic effects of *L. plantarum* in waterfowl—particularly Muscovy ducks, which are highly susceptible to APEC—remain insufficiently characterized. Furthermore, no studies have evaluated whether a naturally fermented-food–derived *L. plantarum* strain can protect ducks from APEC-induced intestinal injury. Given that ZG-7 has previously shown potent inhibitory activity against the Gram-negative pathogen R. anatipestifer, which shares key outer-membrane features and mucosal injury patterns with APEC, we hypothesized that ZG-7 may possess broader antagonistic potential against Gram-negative enteric pathogens. Specifically, we proposed that ZG-7 could alleviate APEC-induced gut injury by enhancing mucin and tight-junction protein expression and by modulating the cecal microbiota. Therefore, this study was designed to elucidate the strain-specific protective mechanisms of *L. plantarum* ZG-7 using an APEC-infected Muscovy duck model.

## 2 Materials and methods

### 2.1 Isolation and purification of bacterial strains

*Lactobacillus plantarum* ZG-7 was originally isolated from naturally fermented pickles collected from a village in Fujian Province, China. The fermentation process was carried out under natural conditions without the addition of starter cultures. The strain was deposited in the China General Microbiological Culture Collection Center (CGMCC, accession no. 7370). Bacteria were isolated from the pickle samples following previously described protocols with minor modifications. Briefly, solid samples were chopped and mixed with sterile phosphate-buffered saline (PBS), and the mixture was homogenized using a blender (Saivier, Wuhan, China). The homogenates were vortexed vigorously, plated onto de Man–Rogosa–Sharpe (MRS) agar (Haibo, Qingdao, China), and incubated anaerobically at 37°C for 48 h. Colonies were subcultured at least three times until purified isolates were obtained. For preparation of bacterial suspensions, cells were harvested by centrifugation at 3000 rpm for 10 min at 4°C, and the pellets were washed and resuspended in sterile saline to a final concentration of 1 × 10^9 CFU/mL. Strain identification was performed by 16S rDNA sequencing. Genomic DNA was extracted using a commercial bacterial genomic DNA extraction kit (Ezup Column, Sangon Biotech, Shanghai, China). The 16S rRNA gene was amplified by PCR, purified, and sequenced by Shanghai Sangya Biotechnology Co., Ltd. Viable cell counts of ZG-7 were quantified by spread-plating 10-fold serial dilutions onto MRS agar and incubating at 37°C for 24 h to ensure a final concentration of 2 × 10^9 CFU/mL for oral administration. All bacterial suspensions were freshly prepared for each experiment. If short-term storage was required (< 2 h), the suspensions were kept at 4°C in the dark, and viability was confirmed at 0, 2, and 4 h to ensure stability (>95% of the initial CFU).

### 2.2 Acid and bile salt tolerance assays

To evaluate the acid and bile tolerance of *Lactobacillus plantarum* ZG-7, overnight cultures were prepared in sterile MRS broth (Haibo, Qingdao, China) at 37°C. Cells were harvested by centrifugation (3000 rpm, 10 min, 4°C), washed twice with phosphate-buffered saline (PBS), and resuspended in fresh MRS broth. For acid challenge assays, bacterial suspensions were diluted 1:100 in MRS broth adjusted to pH 2.0, 3.0, or 4.0. Bile salt tolerance was determined by inoculating suspensions into MRS broth supplemented with 0.1%, 0.3%, 0.5%, or 1% (w/v) bile salts (Yuanye Biotechnology Co., Ltd., Shanghai, China). All cultures were incubated at 37 °C with agitation at 150 rpm. Bacterial survival and growth under acidic or bile salt conditions were monitored exclusively by measuring optical density at 600 nm (OD600) at 0, 6, 12, 18, and 24 h. OD600 values served as indicators of ZG-7 proliferation and tolerance under each stress condition.

### 2.3 Antibacterial activity of *Lactobacillus plantarum*

The antibacterial activity of the cell-free supernatant of *Lactobacillus plantarum* ZG-7 against avian pathogenic *Escherichia coli* O78 was assessed using the Oxford cup assay. The O78 strain was obtained from the Institute of Animal Husbandry and Veterinary Medicine, Fujian Academy of Agricultural Sciences (FAAS, China). ZG-7 was cultured in MRS broth for 24 h, and the culture was centrifuged at 10,000 rpm for 5 min. The supernatant was filtered through a 0.45 μm membrane, and a portion was adjusted to pH 7.0 to serve as the control (CON). The pathogenic strain was spread on LB agar plates, and Oxford cups were placed on the surface. The wells were filled with ZG-7 supernatant at concentrations of 1 × 10^8 CFU/mL and 1 × 10^9 CFU/mL. Plates were incubated at 37°C for 8 h, after which inhibition zones were measured.

### 2.4 Animal experiment

A total of 40 healthy one-day-old Muscovy ducks (equal numbers of males and females) were obtained from Zhangzhou Changlong Animal Husbandry Co., Ltd. (Fujian, China) and randomly allocated into five groups of equal initial body weight. Randomization was performed using a random-number table to ensure unbiased allocation of animals to experimental groups. All birds were housed separately under identical conditions and fed a basal diet. Ducks were reared in two-tier wire cages with ad libitum access to feed and water. The lighting schedule consisted of 24 h continuous light from days 1 to 7, followed by 23 h light/1 h dark thereafter. Room temperature was maintained at 32°C during the first week and gradually decreased to 25°C by day 15. No antibiotics, coccidiostats, or other microbiota-altering agents were administered throughout the experiment. A corn–soybean basal diet was formulated according to the nutritional recommendations of the National Research Council (NRC, 1994) and the Chinese Feeding Standard for Meat Ducks (NY/T 2122-2012) to meet the nutrient requirements of Muscovy ducks. The composition and nutrient levels of the basal diet are provided in **Supplementary Table 1**.

**Figure 1:**
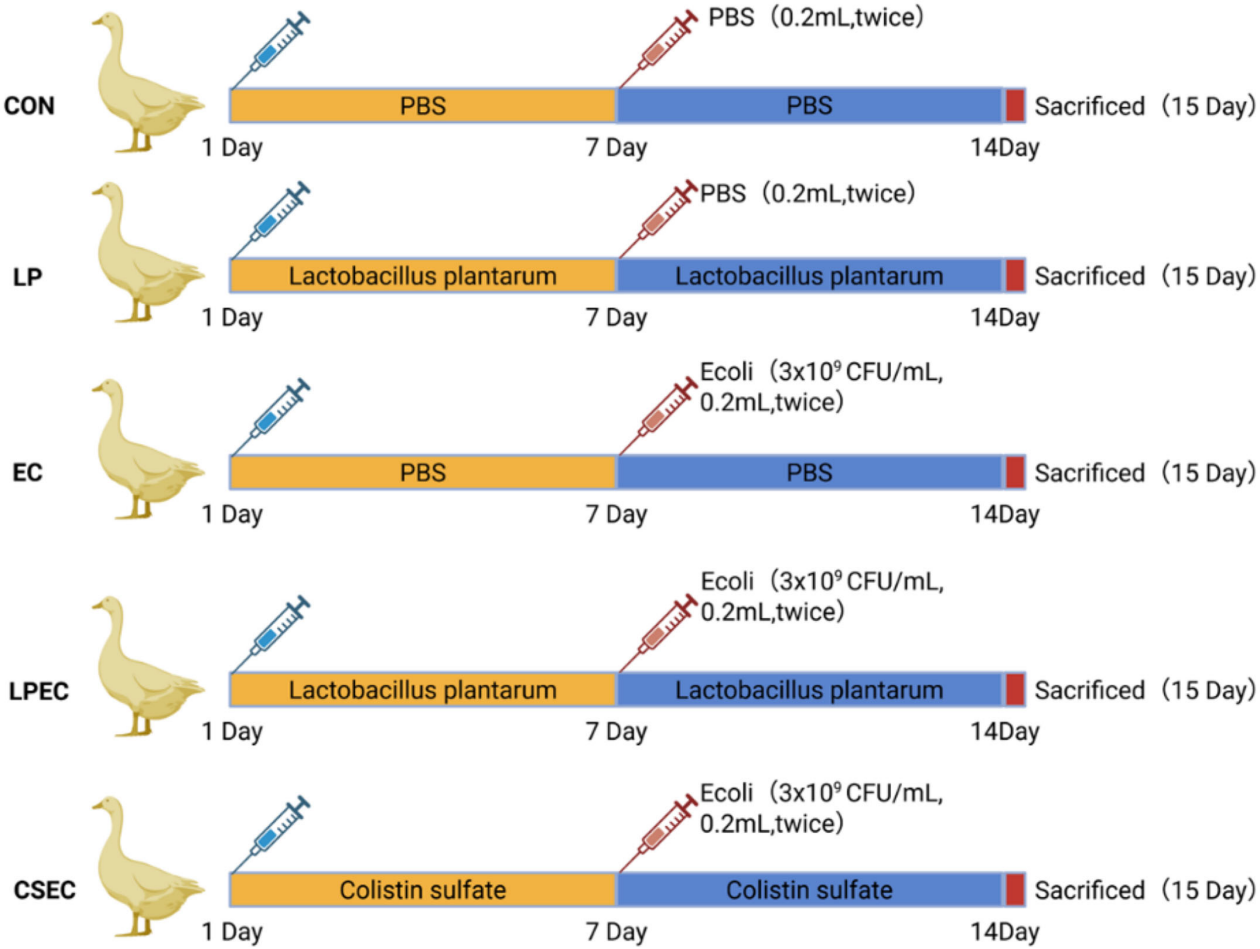
Workflow of the experiment.

The control group (CON) received PBS without *E. coli* O78 challenge; the infection group (EC) received PBS and was orally challenged on day 7 with two doses of *E. coli* O78 suspension (3 × 10^9 CFU/mL, 0.2 mL each) administered 8 h apart; the probiotic group (LP) was administered 0.5 mL of Lactobacillus plantarum ZG-7 (2 × 10^9 CFU/mL) daily; the probiotic + challenge group (LPEC) received the same probiotic treatment daily and was orally challenged on day 7 with two doses of *E. coli* O78 suspension (3 × 10^9 CFU/mL, 0.2 mL each) administered 8 h apart; and the colistin + challenge group (CSEC) was administered 0.5 mL of colistin sulfate solution (200 mg/mL) daily and was orally challenged on day 7 with two doses of *E. coli* O78 suspension (3 × 10^9 CFU/mL, 0.2 mL each) administered 8 h apart. The experimental workflow is illustrated in **Figure 1**. All histopathological evaluations (HE, AB-PAS, immunofluorescence) and microbial analyses were conducted by independent investigators who were blinded to the treatment groups throughout the study.

### 2.5 Ethics statement

All experimental procedures involving animals were conducted in accordance with institutional and national guidelines for the care and use of laboratory animals. The protocol was reviewed and approved by the Animal Ethics Committee of the Animal Husbandry and Veterinary Research Institute, Fujian Academy of Agricultural Sciences (Approval No. MYLISC2024-012). All efforts were made to minimize animal stress and discomfort.

### 2.6 Sample collection

On day 15, ducks were fasted for 12 h and then euthanized by cervical dislocation. The thymus, spleen, liver, and bursa of Fabricius were aseptically excised, rinsed to remove surface blood, weighed, and recorded. A 1.5 cm segment of the mid-jejunum was collected, gently cleared of digesta, washed with physiological saline, and immediately fixed in 10% neutral buffered formalin for histomorphological analysis. In addition, portions of cecal contents were rapidly frozen in liquid nitrogen and stored at –80 °C for subsequent analyses.

### 2.7 Measurement of body weight gain and immune organ index

The body weight of each duck was recorded daily, and body weight gain was calculated accordingly. Following sample collection, the thymus, spleen, and bursa of Fabricius were excised and weighed. The relative immune organ index was calculated using the formula:


Organ index=organ weight (mg)/body weight (g)

### 2.8 Intestinal morphological analysis

Jejunal tissue samples were fixed in 4% (w/v) paraformaldehyde buffer for at least 48 h, dehydrated through a graded ethanol series, and cleared in xylene. The tissues were embedded in paraffin, sectioned at a thickness of 5 μm, and stained with hematoxylin and eosin (H&E). The sections were examined and photographed under a light microscope (Nikon Eclipse CI, Nikon Instruments Inc., Tokyo, Japan). Villus height and crypt depth of the jejunum were measured using CaseViewer software (version 2.0, Budapest, Hungary).

### 2.9 Alcian blue–periodic acid–Schiff staining

Jejunal tissue sections were stained using the Alcian blue–periodic acid–Schiff (AB-PAS) method. Fixed sections were dehydrated sequentially in graded ethanol for 2 min each, oxidized in periodic acid solution for 5 min, and rinsed in warm water for 10 min. The slides were then counterstained with Coleman’s Schiff reagent for 10 min. The stained sections were examined and photographed under a light microscope at 100× magnification.

### 2.10 Immunofluorescence staining

Paraffin-embedded jejunal tissue sections were deparaffinized, rehydrated, and subjected to antigen retrieval in sodium citrate buffer, followed by three washes with PBS (5 min each). The sections were then blocked with 5% bovine serum albumin (BSA; Solarbio, Beijing, China) for 30 min at room temperature. Primary antibodies against ZO-1, MUC2, and Occludin (1:500; Bioss, Beijing, China) were applied, and sections were incubated overnight at 4°C. After PBS washing, sections were incubated with FITC-conjugated secondary antibodies (1:200; Solarbio, Beijing, China) for 1 h at room temperature. Finally, nuclei were counterstained with DAPI (Solarbio, Beijing, China), and images were acquired using a fluorescence microscope (Olympus IX53, Tokyo, Japan).

### 2.11 Cecal microbiota sequencing

Total DNA from cecal contents was extracted using the FastDNA SPIN Kit for Feces (MP Biomedicals, Santa Ana, CA, USA). DNA concentration and purity were determined using a spectrophotometer, and integrity was assessed by agarose gel electrophoresis. The V3–V4 region of the bacterial 16S rRNA gene was amplified with universal primers 341F (5’-CCTACGGGNGGCWGCAG-3’) and 805R (5’-GACTACHVGGGTATCTAATCC-3’), and primer specificity was verified by gel electrophoresis. Purified PCR products were subjected to Illumina NovaSeq 6000 sequencing (paired-end 2 × 250 bp), with each sample generating more than 30,000 raw reads. Sequence processing followed the procedures described in our previous work (11), with additional quality control steps implemented in this study. Raw reads were imported into QIIME2 and processed with the DADA2 pipeline for quality filtering, denoising, chimera removal, and amplicon sequence variant (ASV) inference. Representative ASVs were assigned taxonomy against the SILVA 138 database. Prior to downstream analyses, feature tables were rarefied to a uniform sequencing depth. Alpha diversity (Simpson index) and beta diversity (Bray–Curtis distance, PCoA, and NMDS) were calculated in QIIME2, and differential taxa were identified using LEfSe with an LDA threshold of 3.5.

### 2.12 Statistical analysis

Statistical analyses were performed using one-way analysis of variance (ANOVA) in SPSS software (version 26.0; IBM, USA), followed by Tukey’s *post hoc* multiple comparisons to evaluate differences among groups. Graphs were generated using GraphPad Prism (version 10.4; GraphPad Software, USA). Results are expressed as mean ± standard error of the mean (SEM), with statistical significance set at *P* < 0.05 (*), *P* < 0.01 (**), and *P* < 0.001 (***).

## 3 Results

### 3.1 Acid and bile salt tolerance

**Figure 2:**
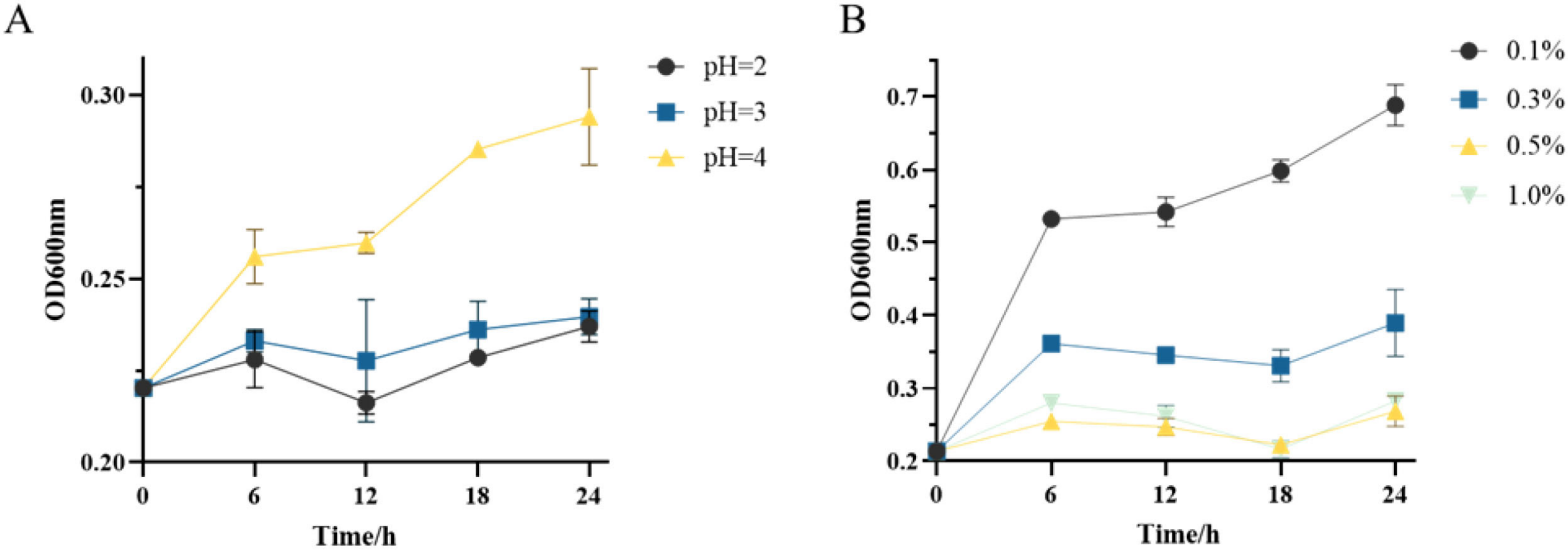
Acid and bile salt tolerance of *Lactobacillus plantarum* ZG-7. **(A)** Growth curves of ZG-7 at different pH values (OD600); **(B)** Tolerance of ZG-7 to various concentrations of bile salts.

As shown in **Figure 2A**, Lactobacillus plantarum ZG-7 was able to survive under acidic conditions ranging from pH 2.0 to 4.0, with continuous growth observed up to 24 h at pH 4.0, indicating strong acid tolerance. The bile salt tolerance assay (**Figure 2B**) demonstrated that ZG-7 maintained growth at bile salt concentrations between 0.1% and 1.0%, with greater proliferation observed at lower concentrations, particularly within the range of 0.1%–0.3%, where growth was most pronounced.

### 3.2 Antibacterial activity of cell-free supernatant of *Lactobacillus plantarum* ZG-7

**Figure 3:**
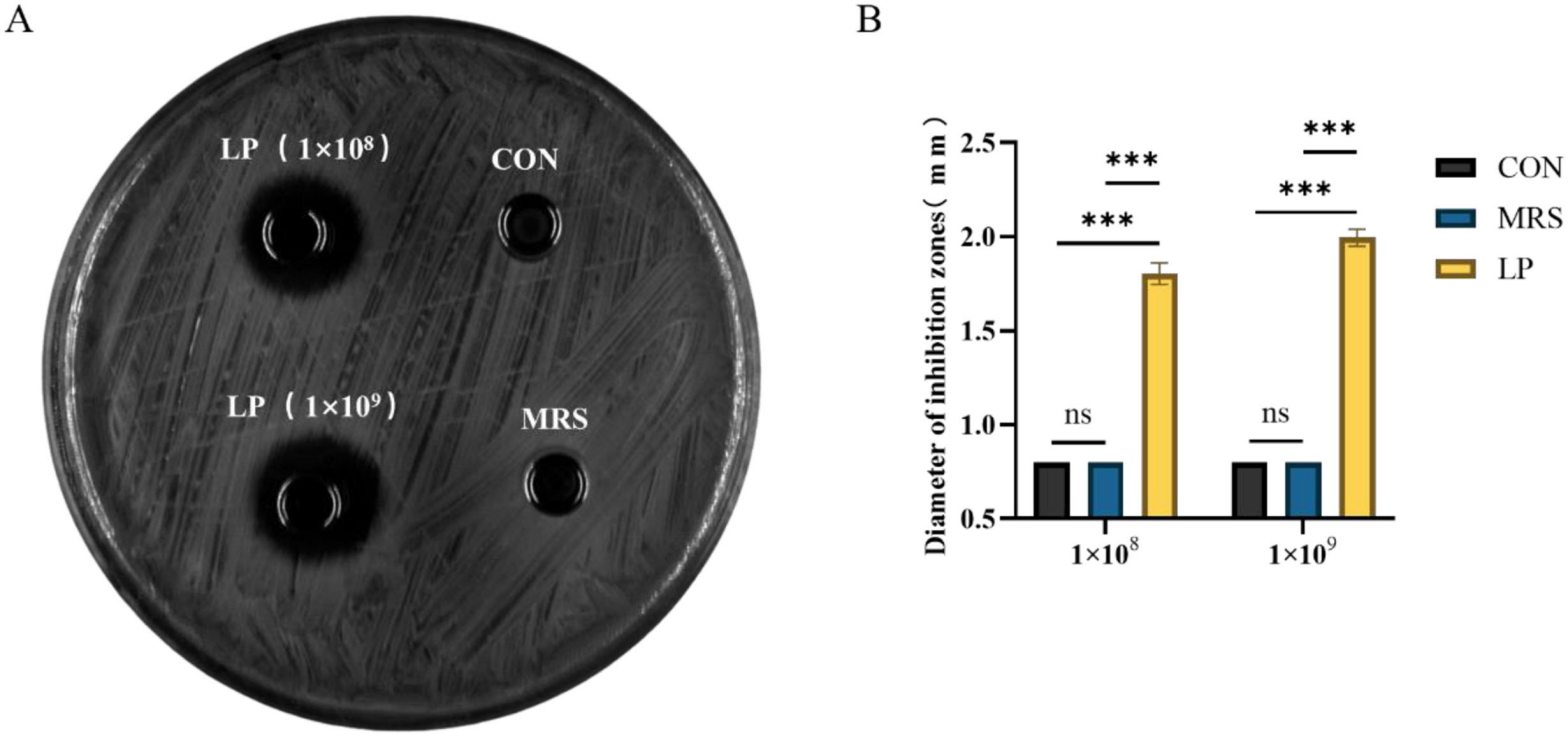
Antibacterial effect of *Lactobacillus plantarum* ZG-7 against *E. coli* O78. **(A)** Antibacterial activity; **(B)** Diameter of inhibition zones. The asterisk (*) indicates a statistically significant difference between the LP group and the CON and MRS groups (P < 0.05).

As shown in **Figures 3A, B**, the cell-free supernatant (LP) of *Lactobacillus plantarum* ZG-7 exhibited significant inhibitory activity against avian pathogenic *Escherichia coli* O78. Moreover, the inhibitory effect was stronger at a bacterial concentration of 1 × 10^9 CFU/mL than at 1 × 10^8 CFU/mL. However, when the pH of the supernatant was adjusted to 7.0, no antibacterial activity was observed.

### 3.3 Effect of *Lactobacillus plantarum* ZG-7 on body weight of Muscovy ducks challenged with APEC

**Figure 4:**
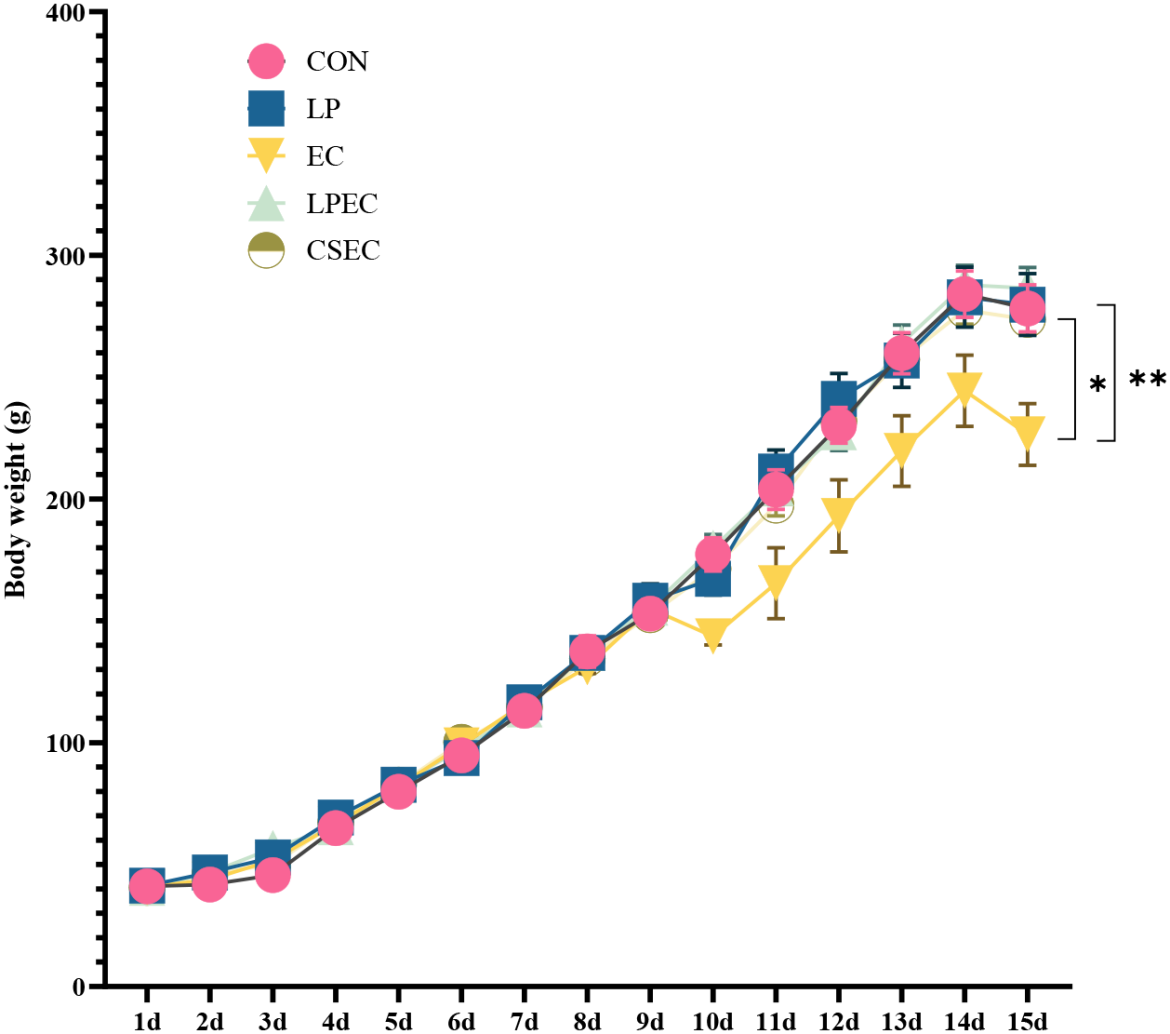
Body weight change curves of Muscovy ducks. On day 15, * indicates a statistically significant difference between the LPEC group and the EC group (P < 0.05), and ** indicates a statistically significant difference between the CON group and the EC group (P < 0.01).

As shown in **Figure 4**, body weight of ducks significantly decreased between days 10 and 15 following *E. coli* challenge. Compared with the EC group, both the probiotic-treated group (LPEC) and the colistin-treated group (CSEC) exhibited significantly higher body weights during this period, with values on day 15 markedly greater than those of the EC group (*P* < 0.05). In addition, the CON and LP groups, which were not challenged with *E. coli*, also showed significantly higher body weights than the EC group; however, their weights were not significantly different from those of the LPEC and CS groups (*P* > 0.05). These findings indicate that *L. plantarum* ZG-7effectively prevented body weight loss in Muscovy ducks caused by APEC infection.

### 3.4 Effect of *Lactobacillus plantarum* ZG-7 on immune organ indices of Muscovy ducks challenged with APEC

**Figure 5:**
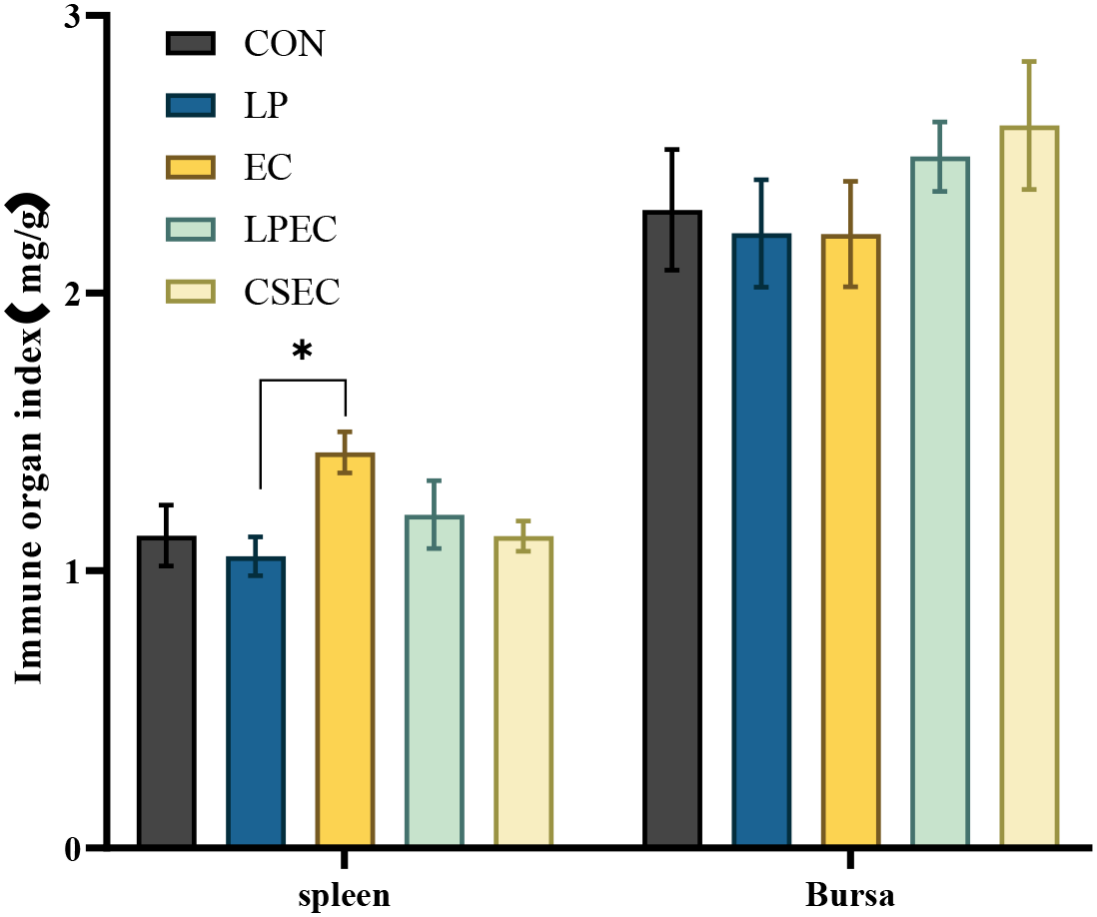
Immune organ indices of Muscovy ducks. Compared with the EC group, the splenic index of the LP group showed a statistically significant difference (P < 0.05).

As shown in **Figure 5**, on day 15, the spleen index of all treatment groups was lower than that of the EC group, while the bursa index was higher. Among the treatment groups, a significant decrease in spleen index compared with the EC group was observed only in the LP group (*P* < 0.05). No significant differences in spleen or bursa indices were detected between the EC group and the LPEC or CSEC groups (*P* > 0.05). These results indicate that APEC challenge markedly affected immune organ indices, while probiotic or antibiotic administration produced only limited modulatory effects within the experimental period.

### 3.5 Effect of *Lactobacillus plantarum* ZG-7on intestinal morphology and goblet cells in Muscovy ducks challenged with APEC

**Figure 6:**
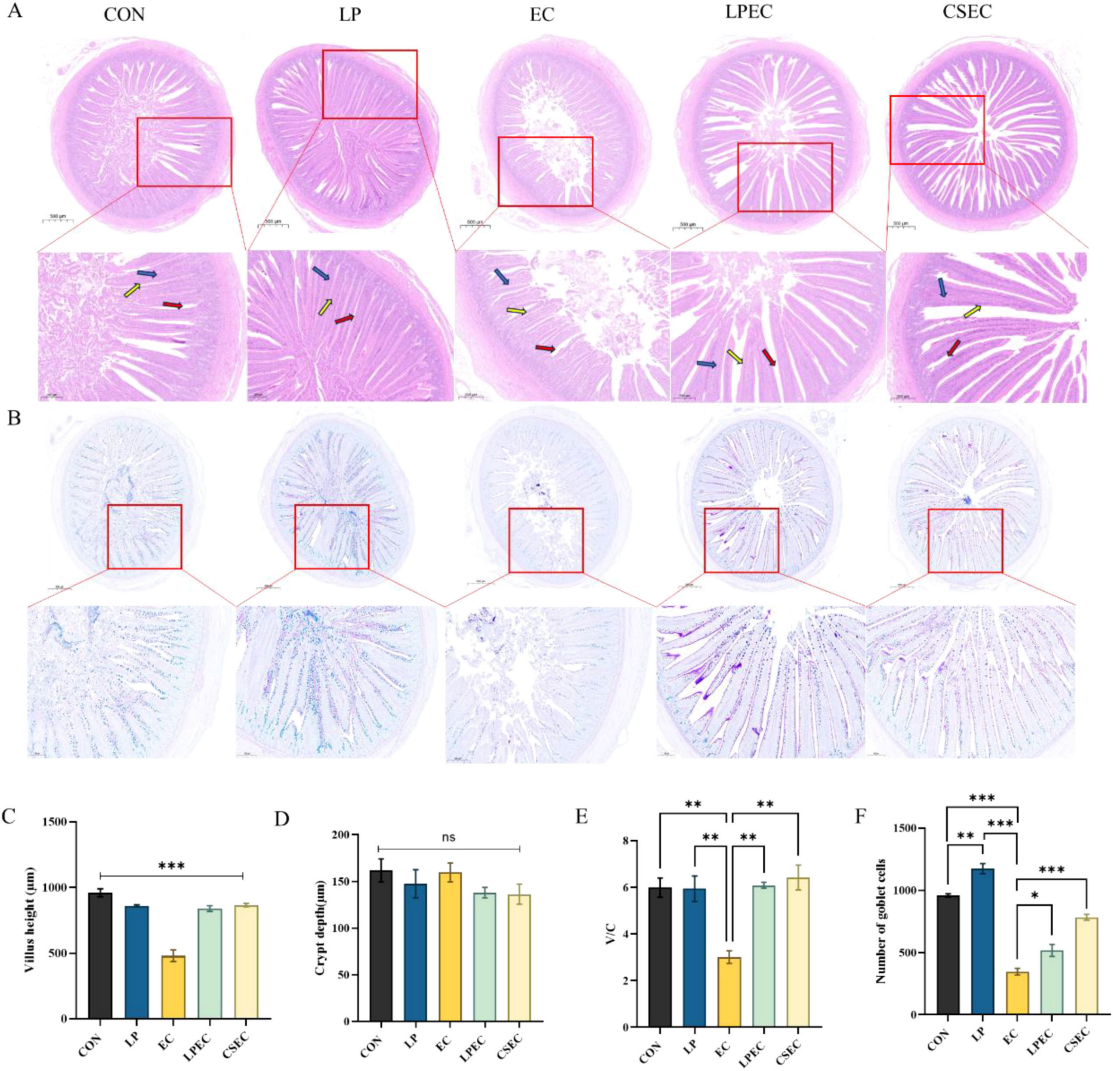
Jejunal morphology and goblet cell counts in Muscovy ducks. **(A)** H&E staining; **(B)** AB-PAS staining; **(C)** villus height; **(D)** crypt depth; **(E)** villus-to-crypt ratio (V/C); **(F)** goblet cell counts. Yellow arrows indicate epithelial cell damage, blue arrows indicate crypt loss, and red arrows indicate gland loss. Magnification: ×40 and ×200. In **C** and **E**, * indicates a statistically significant difference in villus height and the V/C ratio, respectively, between the EC group and each of the other groups . In **F**, * indicates a statistically significant difference in goblet cell counts between the EC group and each of the other groups.

As shown in **Figure 6A**, compared with the CON group, ducks in the EC group exhibited extensive inflammatory cell infiltration in the jejunal mucosa and submucosa, accompanied by epithelial cell disruption and loss of crypts and glands. Following treatment with *Lactobacillus plantarum*, these pathological changes were alleviated to varying degrees, with markedly reduced inflammatory infiltration and largely preserved epithelial integrity. AB-PAS staining (**Figure 6B**) further demonstrated that, relative to the CON group, ducks in the EC group showed a decreased number of goblet cells and reduced mucus secretion, whereas probiotic treatment increased villus length, enhanced goblet cell abundance within the mucosal epithelium, and restored production of blue-stained mucus. Quantitative analysis (**Figures 6C–E**) revealed that villus height was significantly reduced in the EC group (*P* < 0.001), crypt depth remained unchanged (*P* > 0.05), and the villus-to-crypt (V/C) ratio was markedly decreased (*P* < 0.01). In contrast, probiotic treatment significantly increased the V/C ratio (*P* < 0.01), suggesting a protective effect of *L. plantarum* ZG-7against APEC-induced jejunal damage. Consistent with these findings, higher-magnification AB-PAS images (**Figure 6F**) showed that probiotic treatment increased goblet cell abundance and restored mucus secretion. These results indicate that *L. plantarum* ZG-7effectively mitigated the loss of goblet cells caused by APEC infection.

### 3.6 Effect of *Lactobacillus plantarum* ZG-7 on the jejunal mucosal physical barrier of Muscovy ducks challenged with APEC

**Figure 7:**
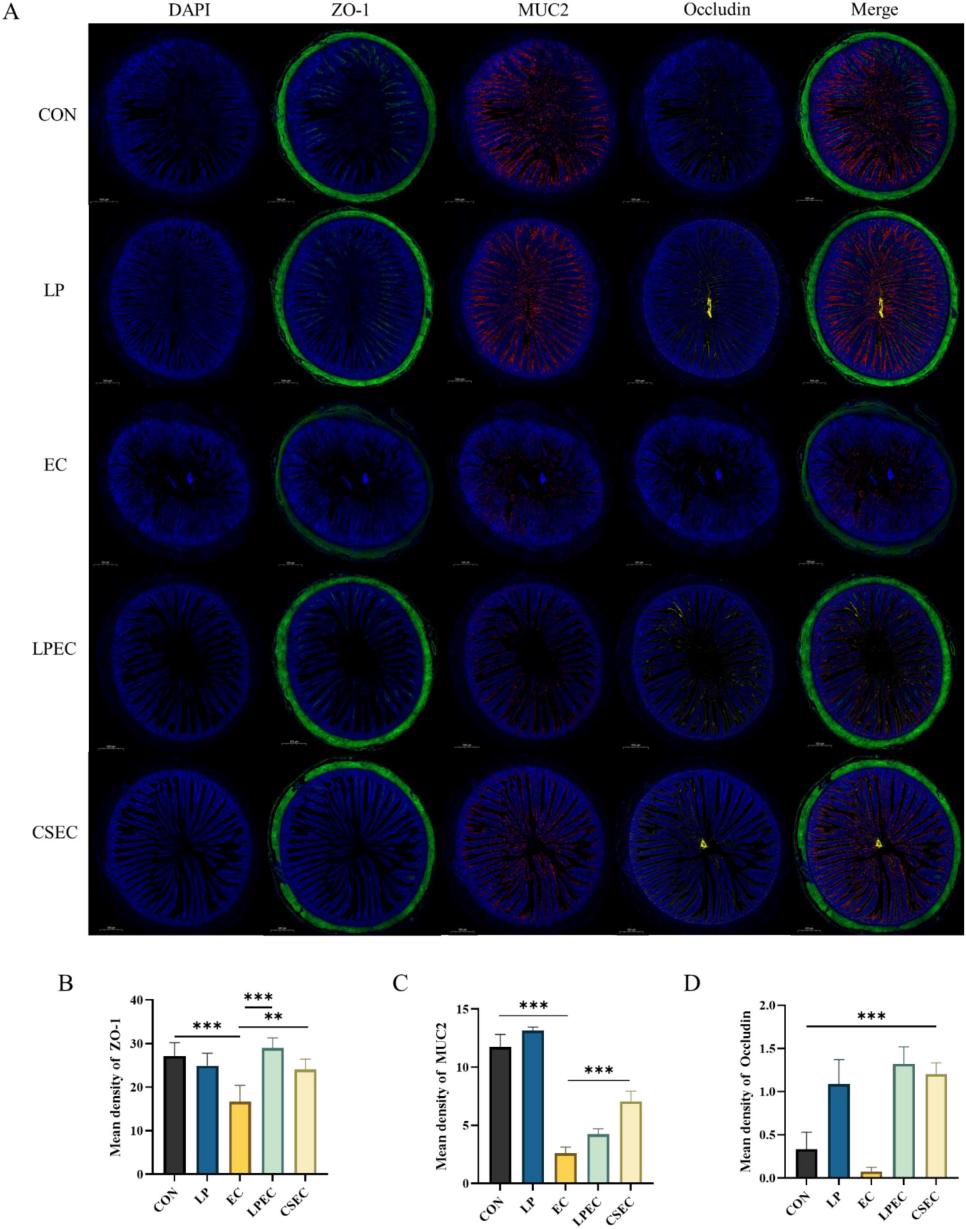
Effect of *Lactobacillus plantarum* on the expression of mucin and tight junction proteins in the jejunum of APEC-infected Muscovy ducks. **(A)** Immunofluorescence analysis of ZO-1, MUC2, and Occludin (magnification ×40); **(B)** ZO-1 expression; **(C)** MUC2 expression; **(D)** Occludin expression. Blue fluorescence indicates nuclei stained with DAPI, green fluorescence indicates ZO-1, red fluorescence indicates MUC2, and yellow fluorescence indicates Occludin. In **B–D**, * indicates a statistically significant difference in the mean fluorescence intensity of ZO-1, MUC2, and Occludin, respectively, compared with the EC group.

As shown in **Figure 7**, supplementation with *Lactobacillus plantarum* ZG-7 increased the expression of jejunal barrier proteins MUC2 and Occludin compared with the CON group, with Occludin showing a significant upregulation (*P* < 0.001). Relative to the EC group, the CSEC group exhibited significantly higher expression of ZO-1 (*P* < 0.01), MUC2 (*P* < 0.001), and Occludin (*P* < 0.001). Similarly, the LPEC group showed increased levels of all three proteins, with ZO-1 (*P* < 0.001) and Occludin (*P* < 0.01) significantly elevated. These results indicate that *L. plantarum* ZG-7 enhances the intestinal mechanical barrier and contributes to maintaining mucosal integrity. As the focus of this study was on the protective mechanisms of *L. plantarum* against APEC-induced intestinal injury, and our earlier results already confirmed the efficacy of colistin in alleviating intestinal damage, no further mechanistic investigations of colistin were pursued.

### 3.7 Effect of *Lactobacillus plantarum* ZG-7 on cecal microbiota richness and diversity in Muscovy ducks challenged with APEC

**Figure 8:**
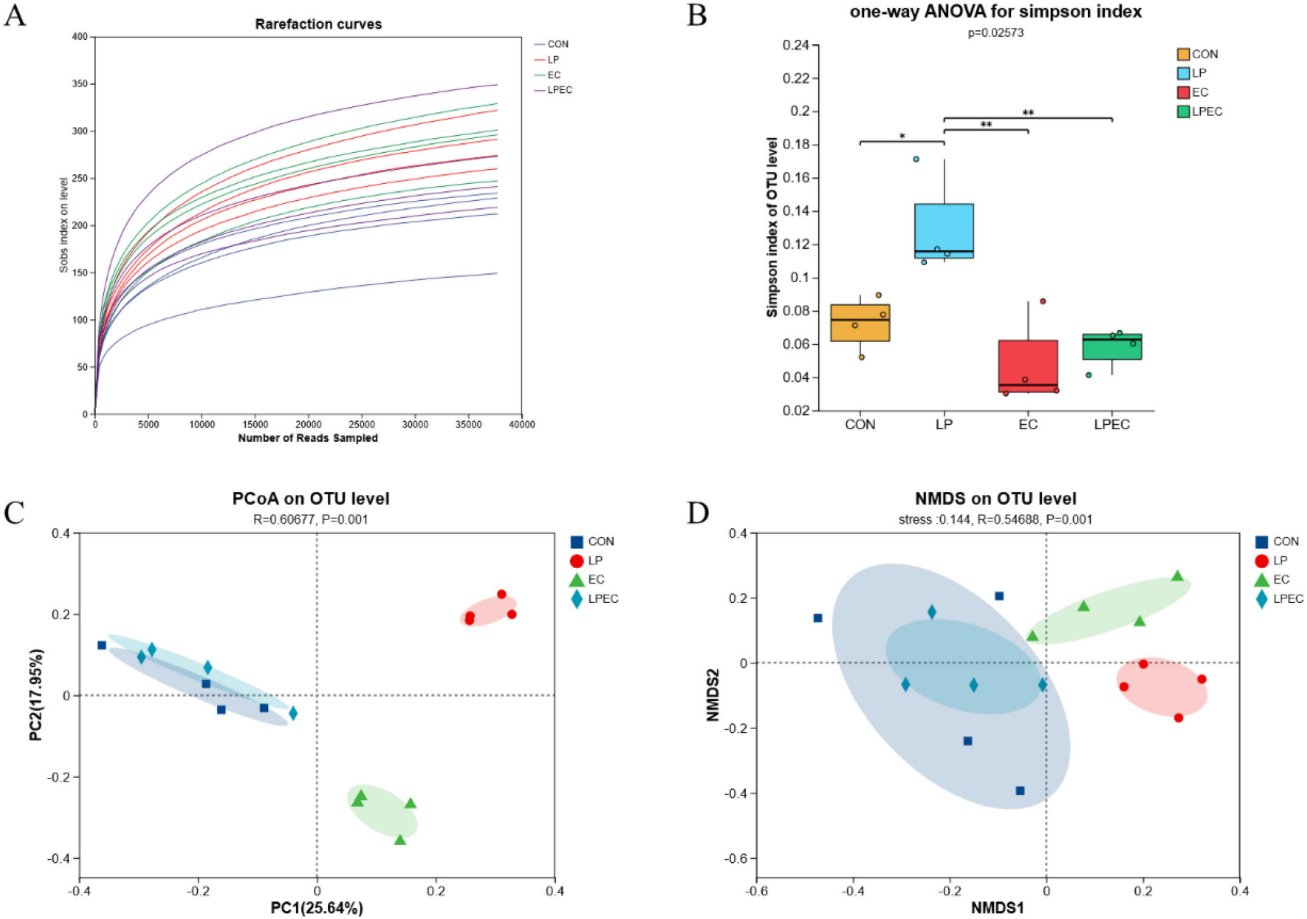
Analysis of intestinal microbiota diversity in Muscovy ducks. **(A)** Rarefaction curves; **(B)** Simpson index; **(C)** principal coordinate analysis (PCoA) based on Bray–Curtis distances of OTUs; **(D)** non-metric multidimensional scaling (NMDS) based on Bray–Curtis distances of OTUs. asterisks (*) indicate statistically significant differences in the Simpson index between the indicated groups (P < 0.05).

To investigate changes in the intestinal microbiota, 16S rRNA gene sequencing of cecal contents was performed, and OTU numbers were analyzed to assess microbial richness. Rarefaction curves (**Figure 8A**) showed that when sequencing depth reached approximately 3,500 reads, the curves plateaued, indicating sufficient sequencing coverage and stable species evenness, suggesting that the sequencing depth was adequate to capture community diversity. Microbial diversity was further evaluated using the Simpson index (**Figure 8B**), where lower values indicate greater diversity. Results revealed that the LP group had significantly reduced microbial diversity compared with the CON, EC, and LPEC groups (*P* < 0.05), while no significant differences were observed among CON, EC, and LPEC groups. To further examine community composition, Bray–Curtis-based principal coordinate analysis (PCoA) and non-metric multidimensional scaling (NMDS) were conducted. As shown in **Figures 8C, D**, microbial communities in the CON and LPEC groups clustered closely, whereas both the LP and EC groups exhibited distinct community structures compared with the CON group.

### 3.8 Effect of *Lactobacillus plantarum* ZG-7 on intestinal microbial composition in Muscovy ducks challenged with APEC

**Figure 9:**
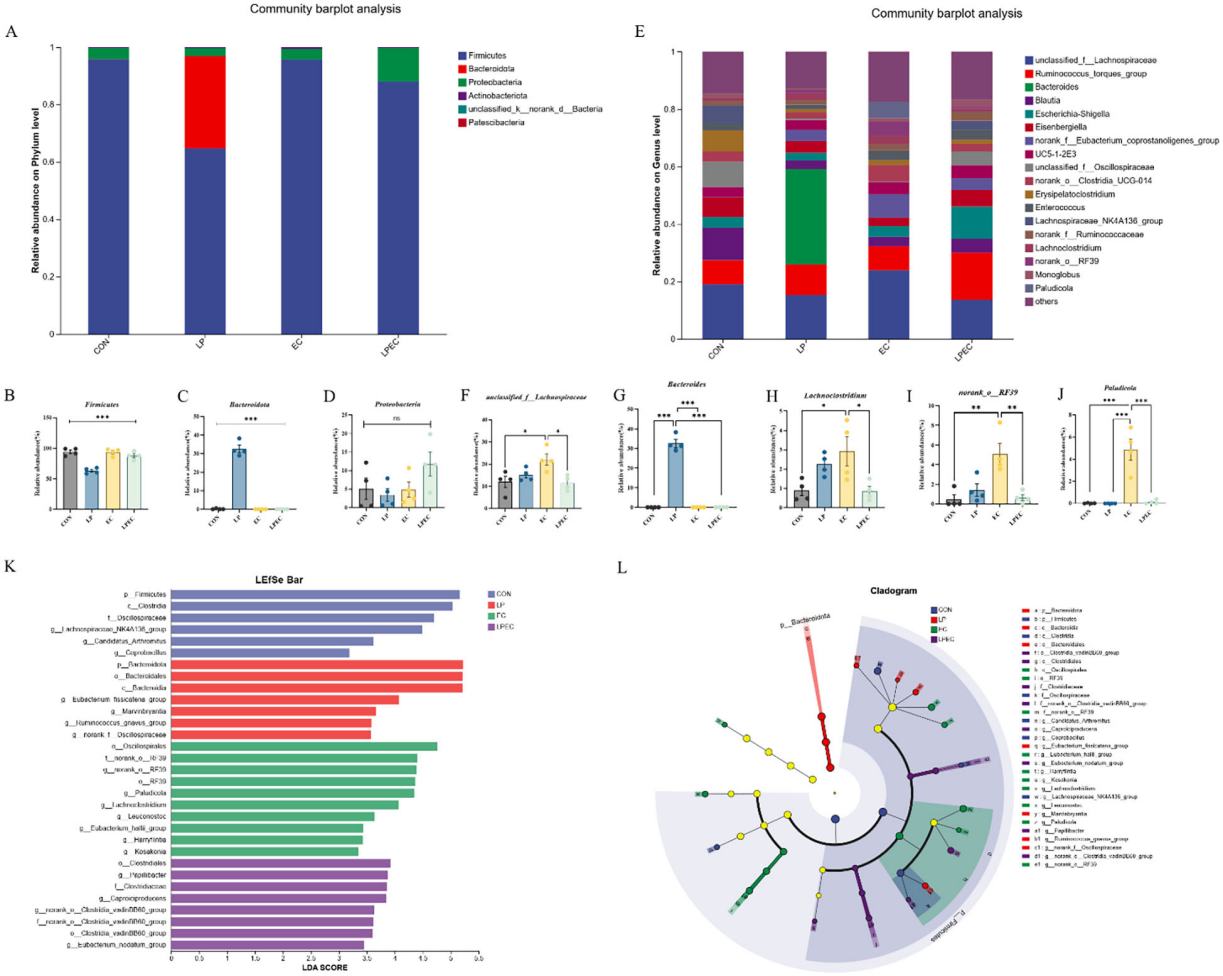
Composition of intestinal microbiota in Muscovy ducks. **(A)** Cecal microbiota composition at the phylum level; **(B)** cecal microbiota composition at the genus level; **(C)** relative abundance of *Firmicutes*; **(D)** relative abundance of *Bacteroidota*; **(E)** relative abundance of Proteobacteria; **(F)** relative abundance of *Unclassified Lachnospiraceae*; **(G)** relative abundance of *Bacteroides*; **(H)** relative abundance of *Lachnoclostridium*; (I) relative abundance of norank RF39 group; (J) relative abundance of *Paludicola*; **(K)** histogram of LDA scores for microbial biomarkers in cecal microbiota; **(L)** cladogram of LEfSe analysis. In **B** and **C**, * indicates a statistically significant difference in the relative abundance of Firmicutes and Bacteroidota, respectively, at the phylum level compared with the EC group . In **F–J**, * indicates statistically significant differences in the relative abundance of unclassified Lachnospiraceae, Bacteroides, Lachnoclostridium, norank RF39 group, and Paludicola, respectively, at the phylum level compared with the EC group.

Taxonomic classification of OTU representative sequences revealed compositional differences across treatment groups at multiple levels. Overall, the cecal microbiota of Muscovy ducks was dominated by *Firmicutes*, *Bacteroidota*, and *Proteobacteria*, with no significant differences among groups (*P* > 0.05) (**Figures 9A–D**). At the genus level, the predominant taxa included *Unclassified Lachnospiraceae*, *Ruminococcus torques* group, *Bacteroides*, *Blautia*, *Escherichia–Shigella*, *Eisenbergiella*, norank *Eubacterium coprostanoligenes* group, UC5-1-2E3, *Unclassified Oscillospiraceae*, Clostridia UCG-014 group, *Erysipelatoclostridium*, *Enterococcus*, *Lachnospiraceae NK4A136* group, *Unclassified Ruminococcaceae*, *Lachnoclostridium*, norank RF39 group, *Monoglobus*, and *Paludicola* (**Figures 9E–J**). Notably, the relative abundances of *Unclassified Lachnospiraceae*, *Lachnoclostridium*, norank RF39 group, and *Paludicola* were significantly higher in the EC group than in the CON group (*P* < 0.05), whereas these taxa were markedly reduced following probiotic treatment (*P* < 0.05). Interestingly, the LP group showed a significantly greater abundance of *Bacteroides* compared with all other groups (*P* < 0.001).

LEfSe analysis with an LDA threshold of 3.5 identified distinct microbial biomarkers among groups (**Figures 9K, L**). The CON group was characterized by enrichment of *Firmicutes*, *Clostridia*, *Oscillospiraceae*, *Lachnospiraceae NK4A136 group*, *Candidatus Arthromitus*, and *Coprobacillus*. The LP group was enriched in *Bacteroidota*, *Bacteroidales*, *Bacteroidia*, *Eubacterium fissicatena group*, *Marvinbryantia*, *Ruminococcus gnavus group*, and norank *Oscillospiraceae*. The EC group was significantly enriched in *Oscillospirales*, norank RF39, *Paludicola*, *Lachnoclostridium*, *Leuconostoc*, *Eubacterium hallii group*, *Harryflintia*, and *Kosakonia*. In contrast, the LPEC group exhibited enrichment of *Clostridiales*, *Papillibacter*, *Clostridiaceae*, norank *Clostridia vadinBB60 group*, and *Eubacterium nodatum group*. Collectively, these results indicate that LP treatment markedly altered the composition of the intestinal microbiota, reshaping the abundance of specific taxa and modulating community structure.

## 4 Discussion

Probiotics must possess the ability to withstand the harsh conditions of the gastrointestinal tract, including exposure to gastric acid and bile salts, which is essential for their survival and colonization. In poultry, the gastric pH typically ranges from 2.0 to 3.0, and only strains that can endure such acidic environments have the opportunity to reach the intestine and exert beneficial effects (12, 13). Bile salts, present in the avian small intestine at concentrations of 0.03%–0.30%, play a critical role in lipid emulsification and digestion; however, probiotics must also tolerate these salts in order to proliferate effectively (14). Thus, strong resistance to acidic conditions and bile salts is a defining characteristic of high-quality probiotics. In our study, *Lactobacillus plantarum* ZG-7 demonstrated the ability to survive and proliferate under both acidic and bile salt stress in a time-dependent manner. Importantly, the ability of probiotics to resist gastrointestinal stressors is not only a prerequisite for colonization, but also underpins their protective role against infectious diseases. Beyond its capacity to persist in the gastrointestinal tract, ZG-7 also exhibited notable antagonistic activity against pathogens, as evidenced by its significant inhibitory effect on avian pathogenic *Escherichia coli* (APEC).

Colibacillosis, particularly caused by APEC, represents one of the most common bacterial infections in poultry and imposes substantial economic losses on the global poultry industry (15). APEC infection not only leads to local and systemic diseases but also significantly impairs growth performance. The emergence of multidrug-resistant APEC strains has further intensified this challenge (16). As a probiotic, *L. plantarum* has been reported to modulate gut microbiota, enhance intestinal barrier integrity, and improve growth performance in poultry, while promoting immune responses and resistance to infection through increasing beneficial taxa and reducing pathogen colonization (8, 17). Consistent with these findings, our study showed that infection with *E. coli* O78 reduced body weight in ducks, whereas treatment with *L. plantarum* ZG-7 significantly alleviated this weight loss. These results align with previous reports demonstrating that APEC infection compromises poultry health and productivity, but supplementation with *L. plantarum* ZG-7can mitigate the detrimental impact of APEC on growth performance (9).

The integrity of the intestinal structure is essential for efficient nutrient absorption and overall host health. Increased villus height enhances the absorptive surface area, whereas crypt depth reflects epithelial renewal and regenerative capacity (18). Continuous migration and turnover of epithelial cells between crypts and villi are critical for maintaining barrier integrity (19). Thus, villus height, crypt depth, and the villus-to-crypt (V/C) ratio are not merely morphological indicators but comprehensive reflections of intestinal health. Previous studies have shown that *E. coli* infection markedly decreases villus height and the V/C ratio while increasing intestinal permeability (20). Goblet cells play a pivotal role in barrier defense through the secretion of the mucin protein MUC2, which forms a protective mucus layer against pathogen invasion and inflammation (21). For instance, supplementation with *Lactobacillus reuteri* in neonatal pigs increased goblet cell abundance and upregulated MUC2 expression, thereby strengthening mucosal barrier function (22). Tight junction proteins such as ZO-1 and Occludin further contribute to epithelial cohesion and barrier stability, and probiotics have been reported to enhance poultry intestinal health by modulating genes related to barrier integrity and immune function (23). In the present study, APEC infection caused severe epithelial shedding, goblet cell loss, and tight junction disruption, leading to villus atrophy, impaired morphology, and reduced nutrient absorption. Remarkably, *Lactobacillus plantarum* treatment mitigated these detrimental effects, partially restoring jejunal architecture and barrier function.

Beyond morphological protection, *L. plantarum* also exerted profound effects on the intestinal microbiota, a key determinant of digestion, immunity, and host performance in poultry. The cecal microbiota contributes to nutrient metabolism, SCFA generation, and immune modulation (24–27). In our study, the LP group exhibited a significant reduction in Simpson diversity, whereas no such difference was observed among the CON, EC, and LPEC groups. Although higher α-diversity is commonly associated with intestinal health, decreased diversity does not universally indicate dysbiosis. Ducks in the LP group showed no signs of intestinal injury—villus morphology, goblet cell abundance, and tight-junction protein expression were comparable to the CON group—suggesting that the lower diversity reflected a compositional shift rather than impaired barrier function. Recent studies also indicate that probiotic-driven niche occupation can transiently lower α-diversity without compromising intestinal health (28). However, the present data do not allow determination of whether this reduction was driven by competitive niche occupation by ZG-7 or other ecological dynamics. Future studies incorporating metabolomics or metagenomic functional profiling are needed to clarify whether the LP-associated community shift corresponds to meaningful metabolic changes (17). β-diversity analyses further revealed that the microbial community structure of the LPEC group more closely resembled the CON group than the EC group, indicating that ZG-7 exerted stronger microbiota-modulating effects under APEC challenge and contributed to restoring microbial community homeostasis.

Analysis of microbial composition revealed that Firmicutes, Bacteroidota, and Proteobacteria were the dominant phyla across all groups, in line with common patterns in poultry. However, genus-level differences were more pronounced. In the EC group, *Unclassified Lachnospiraceae*, *Lachnoclostridium, norank RF39 group*, and *Paludicola* were significantly enriched, taxa often associated with intestinal inflammation and barrier dysfunction (29). Probiotic intervention effectively reduced their relative abundance. Conversely, the LP group displayed an increased abundance of *Bacteroides*, a genus often linked to polysaccharide degradation and SCFA biosynthetic potential. While this enrichment suggests altered functional potential in the microbiota, SCFA involvement cannot be inferred in the absence of direct metabolite measurements; recent poultry studies emphasize that compositional shifts do not always correspond to functional metabolic output (30). LEfSe analysis further confirmed that the EC group was enriched with inflammation-associated taxa, whereas LP supplementation promoted taxa functionally related to carbohydrate metabolism. Collectively, these findings indicate that *L. plantarum* ZG-7 can suppress pathogen-associated bacteria while promoting beneficial taxa under APEC challenge, thereby contributing to the restoration of intestinal microbial homeostasis.

We previously demonstrated that the cell-free supernatant of *Lactobacillus plantarum* ZG-7 exhibits strong antimicrobial activity and induces intestinal antimicrobial defense in Muscovy ducks (10). These findings suggest that short-term exposure to ZG-7 or its metabolites does not cause detectable intestinal toxicity in ducks. Although the present study further supports the short-term safety of ZG-7 under both healthy and APEC-challenged conditions, it does not address long-term colonization stability, potential cumulative effects, or host–microbe interactions beyond the acute phase. Therefore, additional longitudinal studies are required to evaluate whether prolonged administration of ZG-7 affects microbial ecology, immune homeostasis, or metabolic function in a sustained manner. Such investigations will be essential for determining the suitability of ZG-7 for continuous use in commercial poultry production.

Finally, although the CSEC group served as a positive control, direct comparison showed that *Lactobacillus plantarum* ZG-7 produced improvements in intestinal morphology and body weight similar to those of colistin sulfate. Recent evidence suggests that certain *Lactobacillus* strains can achieve protective efficacy comparable to antibiotics in poultry infection models (31). This supports the potential of *Lactobacillus plantarum* ZG-7 as a promising non-antibiotic alternative. Further studies comparing functional immune responses and microbial metabolites between probiotic and antibiotic treatments will be required to strengthen this conclusion.

## 5 Conclusion

This study demonstrated that *Lactobacillus plantarum* ZG-7 tolerates acidic and bile salt conditions, exhibits strong antibacterial activity against E. coli O78, and provides protective effects in an APEC-infected duck model. *Lactobacillus plantarum* ZG-7 alleviated body-weight loss, restored intestinal morphology, enhanced mucin and tight-junction protein expression, and modulated the cecal microbiota by suppressing inflammation-associated taxa and enriching beneficial *Bacteroides*. These findings highlight *Lactobacillus plantarum* ZG-7 as a promising probiotic candidate for supporting intestinal health and reducing dependence on antibiotics in poultry production.

## Data Availability

The data presented in this study have been deposited in the NCBI repository under the accession number PRJNA1400783.
