# Supplementary material for: Effects of Lactobacillus plantarum ZG-7 on the intestinal barrier and intestinal flora of Muscovy ducks infected with avian pathogenic Escherichia coli
Source: Front Immunol. 2026 Jan 16;16:1701722. doi: 10.3389/fimmu.2025.1701722 (PMC12855469; doi:10.3389/fimmu.2025.1701722)
Supplement: Supplementary file 1 [file DataSheet1.pdf]

Supplementary Material

Effects of *Lactobacillus plantarum* ZG-7 on the intestinal barrier and intestinal flora of Muscovy ducks infected with avian pathogenic *Escherichia coli*

Song Peng[1], Bilin Xie[2], Guiheng Mei[3], Yaxiong Ma[4], Xin Lin[3], Mengshi Zhao[1], Fengqiang Lin[1], Zhaolong Li[1*]

[1] Institute of Animal Husbandry and Veterinary Medicine of Fujian Academy of Agricultural Sciences, Fuzhou 350013, China

[2] Putian Institute of Agricultural Sciences, Putian 351144, China.

[3] Key Laboratory of Animal Pathogen Infection and Immunology of Fujian Province, College of Animal Sciences, Fujian Agricultural and Forestry University, Fuzhou 350002, China.

[4] Key Laboratory of Traditional Chinese Veterinary Medicine and Animal Health in Fujian Province, College of Animal Sciences, Fujian Agriculture and Forestry University, Fuzhou 350002, PR China.

*** Correspondence:**
Zhaolong Li
497377512@qq.com

Supplementary Tables

Table 1 The composition and nutrient levels of the basal diets (as fed- basis, %)

Ingredient	Content	Nutrient level[2]	Content
Corn	61.80	AME (MJ/kg)	11.69
Soybean meal	26.00	Crude Protein	20.00
Wheat bran	4.00	Calcium	1.25
Fish Meal	4.20	Available phosphorus	0.70
Limestone	1.22	Crude Fiber	2.8
DL- Methionine	0.13	Lysine	1.05
NaCl	0.20	Methionine	0.45
$CaHPO_4$	0.95	Methionine + cysteine	0.80
Premix[1]	1.50		
Total	100.00		

[1]Provided per kilogram of diet: vitamin A, 8000 IU; vitamin D_3, 3000 IU; vitamin E, 20 IU; vitamin K_3, 2.0 mg; vitamin B_1, 4.0 mg; vitamin B_2, 3.6 mg; vitamin B_6, 4.0 mg; vitamin B_{12}, 0.02 mg; pantothenic acid, 11.0 mg; folic acid, 1.0 mg; biotin, 0.15 mg; nicotinic acid, 10.0 mg; antioxidant 100.0 mg; Fe ($FeSO_4 \cdot H_2O$), 80.0 mg; Cu ($CuSO_4 \cdot 5H_2O$), 10.0 mg; Mn ($MnSO_4 \cdot H_2O$), 80.0 mg; Zn ($ZnSO_4 \cdot H_2O$), 75.0 mg; Se (Na_2SeO_3), 0.3 mg; I (KI), 0.4 mg. [2] Crude protein, calcium and available phosphorus were determined based on triplicate measurements, and the others were calculated values. AME: apparent metabolizable energy.
